# Novel Patterns in High-Resolution Computed Tomography in Whipple Pneumonia

**DOI:** 10.3201/eid3005.231130

**Published:** 2024-05

**Authors:** Hui Li, Jiajia Wu, Xiaojun Mai, Wan Zeng, Shuping Cai, Xiuji Huang, Chunxia Zhou, Jin Li, Qin Jiang, Chunliu Lai, Canmao Xie

**Affiliations:** The Seventh Affiliated Hospital, Sun Yat-sen University, Shenzhen, China

**Keywords:** Whipple disease, Whipple pneumonia, pneumonia, high-resolution computed tomography, bacteria, respiratory infections, Tropheryma whipplei, China

## Abstract

With the use of metagenomic next-generation sequencing, patients diagnosed with Whipple pneumonia are being increasingly correctly diagnosed. We report a series of 3 cases in China that showed a novel pattern of movable infiltrates and upper lung micronodules. After treatment, the 3 patients recovered, and lung infiltrates resolved.

Whipple pneumonia is a rare, chronic, multiorgan disease, with symptoms including arthritis, diarrhea, and weight loss. Diagnosis is traditionally confirmed by a histologic examination of a small bowel biopsy (*1*). The causative pathogen is *Tropheryma whipplei* bacteria, initially identified from the aortic valve of an endocarditis patient in 2000 (*2*). The bacterium was successfully cultured again in 2012 by using a sample of bronchoalveolar lavage fluid (BALF) from a pneumonia patient with an acute pulmonary infection (*2*). By using special culture systems, laboratories can grow positive staining or immunofluorescence detectable bacteria within a macrophage or fibroblast cell in 40–60 days. Metagenomic next-generation sequencing (mNGS) is a useful tool for diagnosis. 

We report 3 patients in China diagnosed with *T. whipplei* pneumonia by using BALF mNGS (Vision Medicals Company, http://www.visionmedicals.com) screening during July 2021–December 2022. The patients had unique radiologic patterns, including upper lung gathering of micronodules forming a galaxy sign, and slightly movable infiltrates before, during, and after treatment.

Patient 1 was a 46-year-old man with a productive cough and a 5-year history of lung abnormality. His lung lesions gradually increased over time, and we found gathering micronodules forming a galaxy sign on the right upper lung (Appendix Figure 1). *T. whipplei* bacteria was the only pathogen we recovered from BALF screened by using mNGS. 

Patient 2 was a 67-year-old man with progressive dyspnea, productive cough, poor appetite, and weight loss. Repeated high-resolution computed tomography (CT) showed gradual increase of diffused micronodules gathering on the upper right lung for 6 months before diagnosis (Figure, panel A). Lesions in the upper right lung also showed movement. After bronchoscopic examination, *T. whipplei* bacteria was the only pathogen we recovered from BALF. Our histologic examination of the lung biopsy showed increased foamy macrophages within the alveolar space and thickened alveolar septal (Figure, panel B); neutrophils were the predominant cell type seen. 

**Figure:**
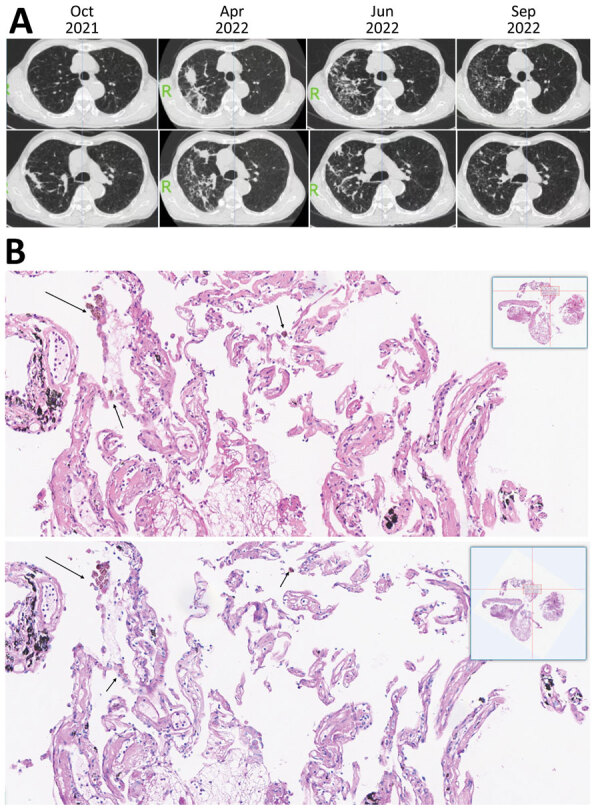
High-resolution computed tomography imaging and histology findings of the lung biopsy from a 67-year-old patient in China who had *Tropheryma whipplei *pneumonia. A) High-resolution computed tomography imaging showing gradual increase of diffused micronodules gathering in the upper right lung 6 months before diagnosis. In October 2021, micronodular and cord-like consolidation were seen on the upper right lung. In April 2022, the lesions were seen changing on both range and pattern and forming movable properties. In June 2022, the lesions were changed and scattered compared with lesions observed in April 2022. In September 2022, lesions were absorbed after 3 months of combined therapy consisting of minocycline and hydroxychloroquine. B) Magnified portion of slide showing histologic findings from the lung biopsy of the patient. The top image shows increased foamy macrophages within alveolar space, thickened alveolar septal and collagen deposition. The top image stain is hematoxylin and eosin staining, with arrows indicating foamy macrophages that have phagocytosed carbon pigment; the bottom image is periodic acid-Schiff staining and is negative for foamy macrophages. Insets show the entire histology slide.

Patient 3 was a 57-year-old man with complaints of cough and chest tightness. We found diffuse ground-glass micronodules in the left upper lung (Appendix Figure 2). We performed mNGS of BALF and found *Cryptococcus* spp. yeast and *T. whipplei* bacteria. We treated the patient with fluconazole. Six months later, the patient was readmitted to our hospital because of chest tightness and dry cough. We repeated mNGS, and *T. whipplei* bacteria was the only pathogen identified.

The lung tissue from all 3 patients was negative for periodic acid-Schiff and anti-acid staining. We performed an enteroscopic examination on the patients 2 and 3; both were negative. We treated the 3 patients with intravenous ceftriaxone (2 g/d) for 2 weeks, then we began combination therapy of minocycline and hydroxychloroquine for an extended period. All 3 patients responded well to treatment, and chest CT showed improvement of lung lesions.

We conducted a literature review for similar cases. We systematically reviewed PubMed for “*T. whipplei*” or “Whipple’s disease” and “pneumonia” for the period July 2021–December 2022. We included literature for analysis if they provided individual patient and imaging data. We defined acute pulmonary infection by classic clinical manifestation and opacity on a chest radiograph or a CT scan. A total of 97 patients with Whipple pneumonia were mentioned. CT findings were available for 14 patients from 7 studies (*2**–**8*). The CT findings included bilateral alveolar consolidation, mass, nodule with cavitation, ground-glass opacity, and diffuse micronodules (Table). Mediastinal lymphadenopathy was described in 1 patient. Therapeutic outcomes were described in 5 patients, and no patients died from pneumonia. Only 1 patient had a comparative chest CT before and after treatment. No patients demonstrated movable lung infiltrates.

**Table:** Categorized data from previously published studies on high-resolution CT findings, symptoms, inflammatory indicators, and immune status in patients with *Tropheryma whipplei* infection*

Study (reference)	Patient age, y/sex	CT findings	Symptoms	Inflammatory indicators	Patient immune status
Fenollar et al. (*2*)	70/F	Diffuse bilateral micronodular involvement, mediastinal lymphadenopathy	Nocturnal sweats, fever, dyspnea, myalgia, arthralgia, diarrhea	Unremarkable leukocyte and CRP levels	Immunocompetent
Stein et al. (*3*)	24/M	Right upper lobe pneumonia and bilateral alveolar condensation	Dyspnea, productive cough, high-grade fever	Elevated leukocyte and CRP levels	HIV
Zhang and Xu (*4*)	26/M	A thick-walled cavity in the left upper lung	Breathing-related chest pain	Unremarkable leukocyte and CRP levels	Immunocompetent
Kelly et al. (*5*)	31/M	Several discrete nodules	Dry cough with progressive weight loss, malaise, poor appetite	Elevated ESR	Immunocompetent
Li et al. (*6*)	39/F	Diffuse bilateral ground glass opacity and consolidation	Coughing, dyspnea, low-grade fever	Elevated neutrophil and CRP levels	Immunocompetent
Canessa et al. (*7*)	60/F	Alveolar consolidation in the left lower lobe, pleural effusion	Diarrhea, progressive dyspnea, dry cough, weight loss	Unknown	Unknown
Lin et al. (*8*)	Mixed	Nodular type: ground glass nodules or solid nodules; pneumonia type: focal or patchy mixed density shadow; other manifestations: cavity, cystic, or pleural effusion.	Respiratory symptoms, weight loss, fever, rare gastrointestinal symptoms	Unremarkable leukocyte and CRP levels	Unknown

*CRP, C-reactive protein; CT, computed tomography; ESR, erythrocyte sedimentation rate.

A 17-year-long retrospective study identified 36 patients with positive PCR results of *T. whipplei* bacteria; of those, 8 patients had pulmonary involvement, and only 3 patients had abnormalities in chest imaging (*9*). Another study showed that 6.1% (88/1,430 samples) of BALF samples were positive for *T. whipplei* bacteria; 58 patients had pneumonia, and *T. whipplei* bacteria was identified as the causative pathogen for 9 patients (*10*). One study analyzed the characteristics of 70 patients positive for *T. whipplei* bacteria in BALF detected by mNGS in which *T. whipplei* was the only pathogen recovered for 20 patients (*8*); in that study, 15 patients received therapy, and 6 patients improved after treatment (*8*). In our study, *T. whipplei* bacteria was the only pathogen in 2 patients and was repeatedly detected in the third patient. In our patients, the infiltrates exhibited movable changes over time before, during, and after treatments. Histologic examination of case 2 showed a collagen and carbon deposition within lung tissue without any history of coal mine exposure, suggesting that *T. whipplei* bacterial infection can cause chronic infection and scar formation, eventually leading to granulomatous-like changes within lung tissue. All 3 patients symptoms improved after receiving the first-line treatment recommendation of minocycline and hydroxychloroquine (*1*).

In conclusion, Whipple pneumonia is increasingly recognized when mNGS is used. We report a relatively unique feature of CT findings in patients with Whipple pneumonia and provide support for choosing combination treatment using minocycline and hydroxychloroquine.

Appendix Additional information about novel patterns in high-resolution computed tomography in Whipple pneumonia.
