# Supplementary material for: Novel Patterns in High-Resolution Computed Tomography in Whipple Pneumonia
Source: Emerg Infect Dis. 2024 May;30(5):1042–5. doi: 10.3201/eid3005.231130 (PMC11060448; doi:10.3201/eid3005.231130)
Supplement: Appendix — Additional information about novel patterns in high-resolution computed tomography in Whipple pneumonia. [file 23-1130-Techapp-s1.pdf]

Article DOI: https://doi.org/10.3201/eid3005.231130

EID cannot ensure accessibility for supplementary materials supplied by authors. Readers who have difficulty accessing supplementary content should contact the authors for assistance.

Novel Patterns in High-Resolution Computed Tomography in Whipple Pneumonia

Appendix

Materials and Methods

DNA extraction and mNGS sequencing

BALF was taken from the patient through broncoscopy. Simply, 1.5mL microcentrifuge tube with 0.6mL sample and 250mL 0.5mm glass bead were attached to a horizontal platform on a vortex mixer and agitated vigorously at 2800–3200 rpm for 30 min. Then 7.2mL lysozyme was added for wall-breaking reaction. 0.3mL sample was separated into a new 1.5mL microcentrifuge tube and DNA was extracted using the TIANamp Micro DNA Kit (DP316, TIANGEN BIOTECH) according to the manufacturer's recommendation. DNA libraries were constructed through DNA-fragmentation, end-repair, adaptor-ligation and PCR amplification. Agilent 2100 was used for quality control of the DNA libraries. Quality qualified libraries were pooled, DNA Nanoball (DNB) was made and sequenced by MGISEQ-2000 platform.

Appendix Table. Metagenomic next-generation sequencing results of 3 patients diagnosed with Whipple's disease between July 2021 to December 2022.

Patients	Age (yrs)	Gender	mNGS copies
case 1	46	male	T.whipplei 557 copies;
case 2	67	male	T.whipplei 58839 copies
case 3–1	57	male	Crytococcus Neofonmans 1101 copies; T. whipplei 87 copies
case 3–2	57	male	T.whipplei 2796 copies;

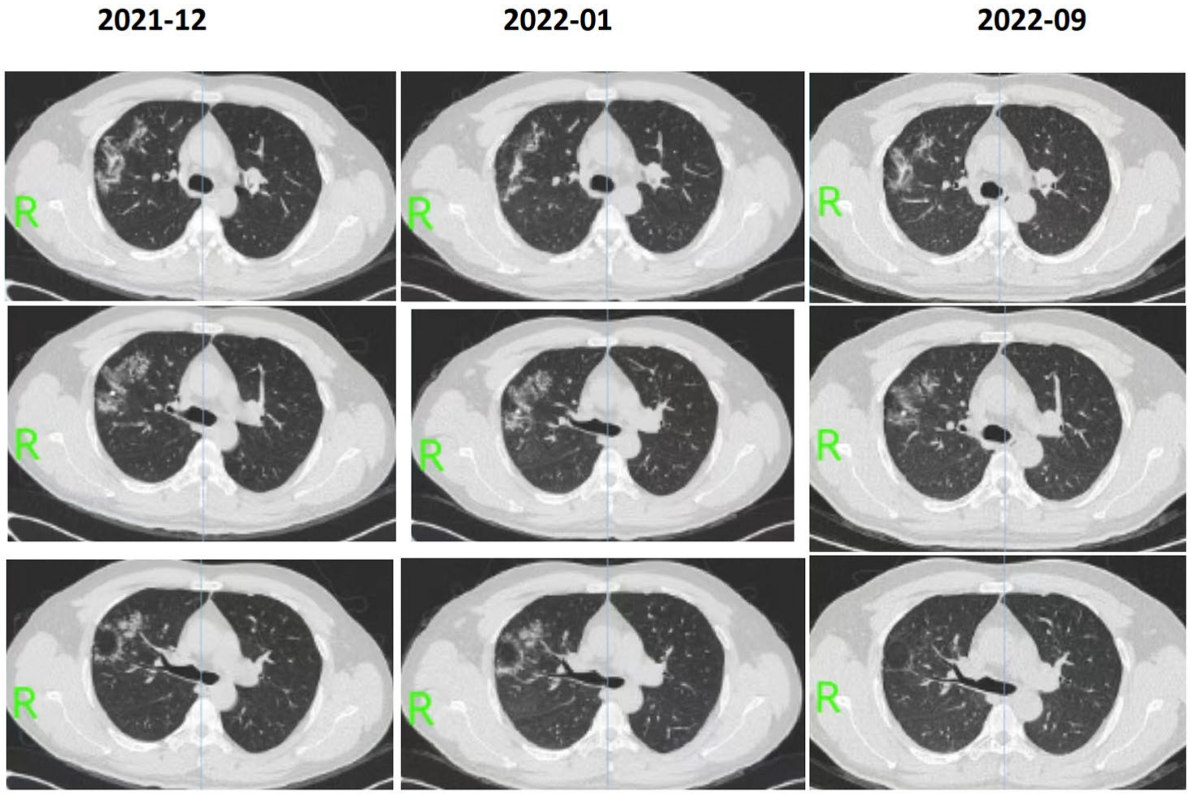

Appendix Figure 1. Case 1, 46 years old patient with lung abnormalities. The 1st row, December-2021, micro-nodular and cord-like consolidation on the upper right lung; the 2nd row, January-2022, the lesions were changed both on range and pattern, and partly assimilated; the 3rd row, September-2022, lesions were changed and partly scattered and partly assimilated, when compared with January-2022. After diagnosis, at the beginning, the patient received anti-whipplei therapy.

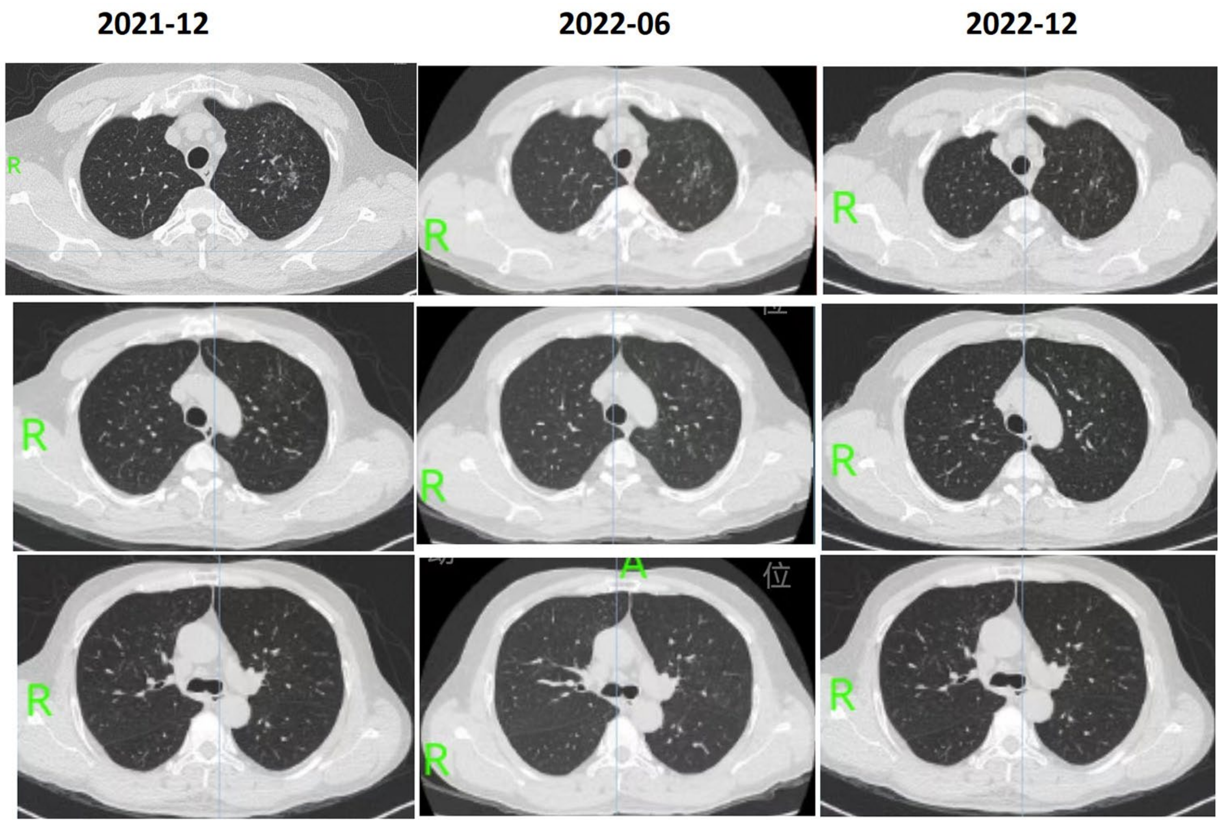

Appendix Figure 2. Case 3, 57 years old male with lung abnormalities. The 1st row, December-2021, micro-nodular scattered predominantly on the upper left lung; the 2nd row, June-2022, the lesions were getting worse on the range and pattern without anti-whipplei's therapy; the 3rd row, December-2022, lesions were partly assimilated and changed after 6 months combined therapy of minocycline and hydroxychloroquine.
